# Dual-Site Transcranial Magnetic Stimulation for the Treatment of Parkinson's Disease

**DOI:** 10.3389/fneur.2019.00174

**Published:** 2019-03-07

**Authors:** Christopher Fricke, Charlotte Duesmann, Timo B. Woost, Judith von Hofen-Hohloch, Jost-Julian Rumpf, David Weise, Joseph Classen

**Affiliations:** ^1^Department of Neurology, University of Leipzig, Leipzig, Germany; ^2^Department of Psychiatry and Psychotherapy, Center for Psychosocial Medicine, University Medical Center Hamburg-Eppendorf (UKE), Hamburg, Germany

**Keywords:** Parkinson's disease, TMS, dual-site, hyperdirect tract, coordinated reset, paired associative stimulation

## Abstract

Abnormal oscillatory activity in the subthalamic nucleus (STN) may be relevant for motor symptoms in Parkinson's disease (PD). Apart from deep brain stimulation, transcranial magnetic stimulation (TMS) may be suitable for altering these oscillations. We speculated that TMS to different cortical areas (primary motor cortex, M1, and dorsal premotor cortex, PMd) may activate neuronal subpopulations within the STN via corticofugal neurons projecting directly to the nucleus. We hypothesized that PD symptoms can be ameliorated by a lasting decoupling of STN neurons by associative dual-site repetitive TMS (rTMS). Associative dual-site rTMS (1 Hz) directed to PMd and M1 (“ADS-rTMS”) was employed in 20 PD patients treated in a blinded, placebo-controlled cross-over design. Results: No adverse events were noted. We found no significant improvement in clinical outcome parameters (videography of MDS-UPDRS-III, finger tapping, spectral tremor power). Variation of the premotor stimulation site did not induce beneficial effects either. A single session of ADS-rTMS was tolerated well, but did not produce a clinically meaningful benefit on Parkinsonian motor symptoms. Successful treatment using TMS targeting subcortical nuclei may require an intervention over several days or more detailed physiological information about the individual brain state and stimulation-induced subcortical effects.

## Introduction

Bradykinesia and tremor impair quality of life in patients suffering from Parkinson's disease (PD), (1). Dopamine replacement therapy is limited by dyskinesia and its symptomatic benefit may be insufficient. Although some motor symptoms can successfully be ameliorated by deep brain stimulation (DBS) of the subthalamic nucleus (2), many patients are reluctant to undergo invasive procedures or are not eligible. In those patients, add-on therapies based on noninvasive brain stimulation techniques may be a promising alternative.

A key element in Parkinsonian pathophysiology is an alteration of information processing within cortico-basal ganglia networks. In particular, the off-motor state has been linked to abnormal beta-oscillatory neuronal activity in a network comprising basal ganglia and motor cortical regions, with the strength of these oscillations being correlated to motor impairment (3–7) and dopamine replacement therapy (8–12). Abnormal beta oscillations within the STN circuitry likely depend on neuronal coupling and synchronized activity. Tass (13) and Popovych and Tass (14) have hypothesized that pathogenic STN oscillatory activity may be dampened using a stimulation protocol tailored to the oscillatory properties which they termed “co-ordinated reset” (CR). In their model, STN neurons may be desynchronized using specific stimulation patterns. Evidence in favor of this approach has been provided in Parkinsonian monkeys (15). An important feature of CR-based DBS is the notion that effects substantially outlasted the duration of the stimulation. This raises the possibility that long-term depression (LTD) has been induced by a Hebbian mechanism in synapses interconnecting STN neurons. In a pilot study the possible efficacy of the method has also been demonstrated in humans (16).

In a systematic review of therapeutic approaches that were based on non-invasive transcranial magnetic brain stimulation (TMS) Chou et al. (17) concluded that TMS was effective in ameliorating bradykinesia when either the primary motor cortex (M1) was stimulated at high (≥5 Hz) frequencies, or more frontal motor regions outside M1 were stimulated at low frequencies (≤ 1 Hz). Although these therapeutic effects might be mediated by induction of changes in cortical excitability another possibility may be a modulatory effect on subcortical structures connected to the cortex via a direct cortico-basal ganglia projection, known as the “hyperdirect tract” (18). This tract has also been discussed as the decisive structure activated by STN-DBS (19–21) and may constitute an interesting target for TMS. Furthermore, evidence exists for a direct short-latency effect of TMS on STN neurons (22, 23) which may have been propagated by the hyperdirect tract. Targeting this tract with TMS may open up a pathophysiologically founded therapeutic stimulation approach targeting pathological oscillatory activity in the STN using TMS. Importantly, as TMS can be timed very precisely it may be able to induce spike-timing dependent plasticity effects in neuronal synaptic connections. Indeed, paired-associative stimulation (PAS) protocols (24, 25) which involve time and location specific activation of neuronal inputs by TMS have been shown to induce LTD-like effects outlasting the intervention for tens of minutes. Plasticity resembling spike-timing dependent plasticity can be induced in cortical neurons by directing timed TMS pulses to two cortical regions (26–28) and subcortically, at the level of the spinal cord, by pairing TMS to M1 with appropriately timed peripheral stimulation (29).

Considering these facts, we aimed to develop a new TMS treatment protocol. We based our protocol on the assumption that different groups of STN neurons may be targeted by TMS mediated by the parts of the hyperdirect tract that originate from premotor and primary motor cortex. As STN neurons oscillate together in the Parkinsonian state, decoupling of these different populations could perhaps be achieved by targeting them with TMS applied in such a way that pulses act on these populations at different times during their oscillatory cycles. We hypothesized that a TMS protocol targeting both primary and premotor areas in a coordinated fashion may achieve this and thus be capable of attenuating pathogenic oscillatory activity in STN neurons which may outlast the stimulation due to LTD-like plasticity effects as shown in CR and PAS protocols.

## Materials and Methods

All procedures were approved by the local Ethics Committee (University of Leipzig, file-no.: 351-13-26082013) and written informed consent was obtained from each participant.

### Patients and TMS Protocol

PD patients were recruited through the outpatient clinic of the Department of Neurology, University Hospital of Leipzig. Inclusion criteria were: age of 18–75 years, Hoehn and Yahr stage 1–3 and a baseline MDS-UPDRS-III of ≥8 points. Exclusion criteria were relevant cognitive impairment (Mini-Mental State Examination < 24), manifest depression (Beck Depression Inventory ≥18), atypical Parkinsonian disorder, other severe illness interfering with safe participation, participations in other studies at the moment of inclusion and known contraindications to TMS (epilepsy, medication with antidepressants, neuroleptics, benzodiazepines, antibiotics, and implanted electrical/metal devices near the head).

**Figure 1:**
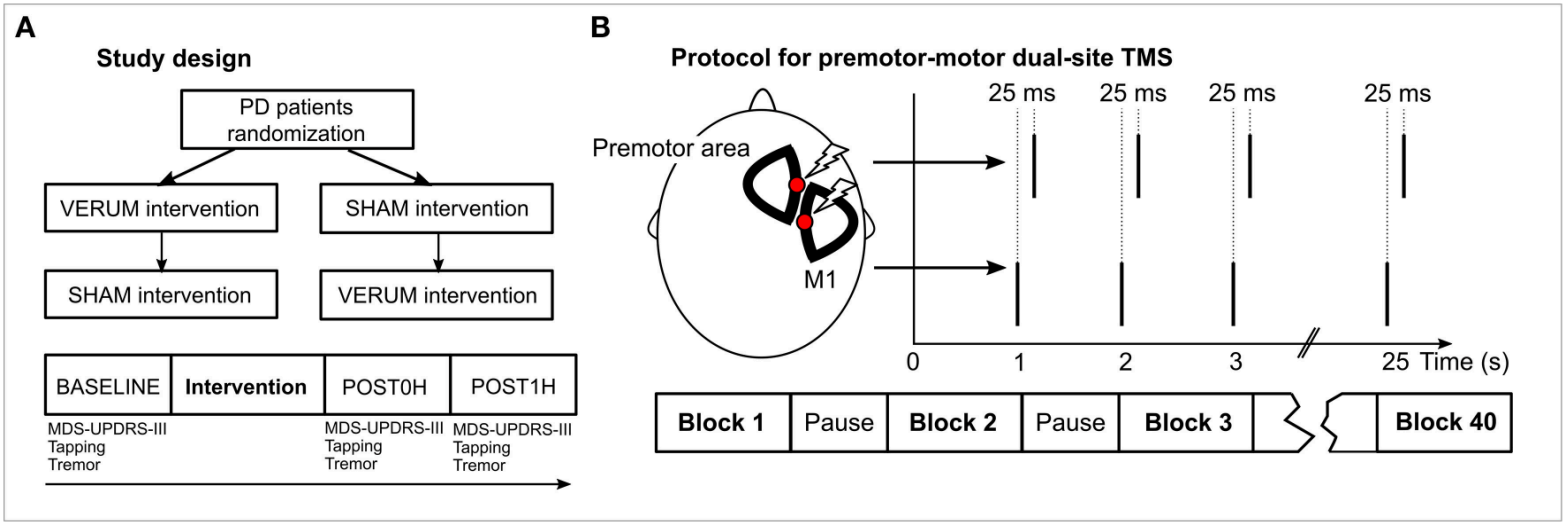
Study design and experimental procedures. **(A)** The general study design is depicted in the panel. PD patients were randomized to receive VERUM or SHAM intervention. A week later each subject received the complementary procedure. At each day of an intervention, motor performance was assessed using MDS-UPDRS-III videography, tapping and tremor analysis prior to the intervention (BASELINE), immediately after the intervention (POST0H) and 1 h later (POST1H). **(B)** During the intervention two stimulation sites (a premotor area and M1) of the hemisphere contralateral to the clinically more affected body side of the patient were stimulated. M1 stimulation was delivered 25 ms before premotor stimulation. Forty blocks of 25 double pulses were applied. Intensity during VERUM stimulation was 95% of the resting motor threshold of the abductor pollicis brevis muscle, 20% during SHAM stimulation.

Patients received two interventions—VERUM (supposedly effective) and SHAM (control)—in a cross-over design following overnight withdrawal of their PD medication. They were randomized to receive either VERUM or SHAM as the first intervention, then they received the complementary procedure at least 1 week later (Figure 1A). Subjects were blinded to the condition and told that “one of two different interventions” would be used. At the day of the intervention, subjects were assessed before (BASELINE), immediately after (POST0H) and 1 h after (POST1H) the intervention (30), comparable to a standardized Levodopa test. Administered tests are detailed below.

We devised an associative dual-site repetitive TMS (“ADS-rTMS”) protocol inspired by CR stimulation (15, 16) and paired associative stimulation (24, 26, 27, 31) protocols. Our TMS protocol included stimulation of a premotor and the primary motor area (M1) (32) to activate distinct regions within STN. We used two coils targeting the hemisphere corresponding to the clinically more impaired body side of the patient (right body side in 12 cases, left side in 8 cases). As a premotor area we primarily targeted the dorsal premotor cortex which we identified physiologically in a localizer experiment (see Supplementary Material). Additionally, we conducted experiments with different premotor sites (see Supplementary Material). One thousand pairs of stimuli were applied in 40 blocks of 25 stimuli each with 5 s pause between each block. Stimuli (double TMS pulses) were delivered at a stimulation frequency of 1 Hz. This frequency ensured rapid completion of the intervention and rendered it unlikely that beneficial stimulation effects were induced by each stimulation site alone (17). Assuming an oscillatory frequency of 20 Hz (8, 33) within the targeted STN, the interstimulus interval (ISI) between premotor and motor TMS pulses was set to 25 ms, with motor cortex stimulation leading premotor stimulation (Figure 1B). This ISI corresponds to a half wave of an oscillation of 20 Hz and, therefore, is supposed to optimally disturb coupled oscillators at this frequency (13). As both stimulation targets are located very close to each other on the scalp, it was not possible to conduct the experiment using conventional figure-of-eight coils. Therefore, we used two custom built D-shaped coils (“Cool-D50 research coils,” external diameter 80 × 59 mm, active cooling) together with two MagPro X100 TMS devices which allowed stimulation of the same cortex area (coils and device MagVenture, Willich, Germany). Despite the different coil geometry, the efficiency of D-shaped coils was comparable to conventional figure-eight coils as indicated by the fact that stimulator outputs for suprathreshold stimulation of M1 were only marginally higher compared to those customarily required with figure-eight shaped coils.

At the start of the TMS intervention we identified the hot spot for stimulation of the abductor pollicis brevis muscle (APB) employing low frequency (< 0.2 Hz) stimulation at multiple sites supposedly overlying M1 while recording MEPs using surface EMG from the APB. We then used threshold hunting (34) to identify the APB resting motor threshold (APB-RMT). This was done for both coils. VERUM stimulation was applied at an intensity of 95% APB-RMT at each coil. For the SHAM stimulation everything was kept identical except we used only 20% APB-RMT. We chose a marginally subthreshold stimulation intensity to stay within safety limits. At 95% APB-RMT corticospinal volleys can be recorded epidurally in patients undergoing spinal surgery (35). This indicates that although this stimulus intensity is insufficient to generate action potentials in spinal motor neurons, it is sufficient to activate corticofugal projection neurons. Additionally, previous studies using TMS to treat PD have successfully used subthreshold intensities (17). We used the BrainSight 2 Neuronavigation (Brain Products, Gilching, Germany) system to control coil positioning. During the intervention subjects were comfortably seated in a reclining position with cushions for their arms and instructed to relax but stay alert and attentive to the tasks. We refrained from testing bradykinesia during the ongoing intervention because LTD-like effects need time to build up and because we aimed to avoid interference by voluntary activity with the intervention.

### Tests and Endpoints

MDS-UPDRS-III, finger tapping performance and tremor activity were recorded for VERUM and SHAM interventions at BASELINE, POST0H, and POST1H as markers for PD motor symptom severity.

#### MDS-UPDRS-III

Global endpoint was improvement in the third part of the Unified Parkinson's Disease Rating Scale of the Movement Disorder Society (MDS-UPDRS-III). The MDS-UPDRS-III was videotaped and later rated by two experienced and certified MDS-UPDRS-III raters (C.F. and T.B.W.) in a randomized order, blinded for condition and time of the recording. As we could not effectively record rigidity on video we excluded this item. We determined the inter-rater agreement using Pearson's and intraclass correlations.

Other clinical, lateralized endpoints were (i) change in a hemibody akinesia score of the treated side (MDS-UPDRS-III items 4–8, range 0–20), (ii) change in a hand akinesia score of the treated side (sum of MDS-UPDRS-III items 4–6, range of 0–12), and (iii) total tremor score (sum of items 15–18) for the treated hand. We hypothesized that VERUM intervention would reduce MDS-UPDRS-III or lateralized MDS-UPDRS-III scores compared to SHAM and/or BASELINE.

#### Finger Tapping Analysis

Subjects performed a finger tapping task during BASELINE, POST0H, and POST1H with tapping performance as a lateralized endpoint. Finger tapping was done on a force transducer (Grass Instruments, West Warwick, USA) which was mounted on a wooden box (size 50 × 30 × 5 cm3) with the level of the transducer slightly above the surface of the box. Subjects were instructed to “tap as quickly as possible” on the force transducer following a go-signal by the experimenter until they were told to stop (after 30 s). The task was performed twice with each hand, starting with the clinically better (untreated) side.

Data pre-processing is described in the Supplementary Material. In order to determine relevant parameters we employed a linear mixed effects model predicting the MDS-UPDRS-III akinesia hand score (sum of items 4–6) from the extracted parameters, which were modeled as fixed effects, while we included the subject specific average tapping force as a random effect. The latter was done to account for individual tapping forces which scale for each subject but are also expected to be different between the clinically worse and the clinically better hand. Significant fixed effect coefficients (*p* < 0.05) were considered relevant parameters for the prediction of the MDS-UPDRS-III hand akinesia score. We hypothesized that VERUM intervention would improve tapping on the treated side. We had to exclude one dataset due to technical issues with the recording devices.

#### Tremor Analysis

Tremor was recorded using triaxial wireless accelerometers (Noraxon, Scottsdale, USA) mounted to either the proximal phalanx of either thumb or index fingers (depending on which finger showed a larger tremor amplitude) of both hands. First, subjects were asked to sit with their hands resting in a semipronated position in their lap (resting tremor). Data was recorded for 30 s, then subjects were given a command to raise both arms and hold them extended in front of them for another 30 s (postural tremor).

Data pre-processing is described in the Supplementary Material. We compared the spectral power of the peak tremor frequency separately for resting and postural tremor analogous to the analysis employed for MDS-UPDRS-III. We hypothesized that tremor power was reduced in response to VERUM stimulation, which was regarded as another lateralized endpoint of the study. We had to exclude one dataset due to movement artifacts.

### Statistical Analysis

We used custom written software in Matlab in combination with the Statistics Toolbox (MathWorks, Natick, USA) for offline data analysis and statistical testing. Presence of normal distributions for outcome parameters was assessed using one-sample Kolmogorov-Smirnov tests, which were non-significant for each parameter. Thus, parametric tests were used for evaluation of all outcome parameters. Primarily, repeated-measures analysis of variance (rmANOVA) in a 2 × 3 within subject design with factors CONDITION (VERUM vs. SHAM stimulation) and TIME (BASELINE vs. POST0H vs. POST1H) or—for baseline-normalized data—in a 2 × 2 within subject design with factors CONDITION (VERUM vs. SHAM stimulation) and TIME (POST0H vs. POST1H) were employed to evaluate effects of VERUM stimulation. We hypothesized that the VERUM but not the SHAM intervention would improve the clinical and technical outcome parameters (MDS-UPDRS-III, tapping performance, tremor power) resulting in a significant CONDITION × TIME interaction and/or a significant main effect for CONDITION. Bonferroni-corrected *post-hoc t*-tests were used to further analyze rmANOVA results. One-sample *t*-tests were used for normalized data to test against unity (with null-hypothesis that test distributions are centered at 1 after normalization). Statistical significance was defined at an alpha level of below 0.05. Average values are usually reported together with their standard deviation in the text while the standard error of the mean is displayed in the figures.

## Results

Twenty PD patients (age 58.5 ± 14.1 years; 15 male, 5 female, Table 1) were included in the experiment (right-handed 16 out of 20). All patients tolerated the procedure well and no adverse events were noted.

**Table 1:** Patient characteristics.

**Subject no**.	**Age (years, range)**	**Disease duration (years)**	**H&Y stage**	**Clinically worse side**	**L-Dopa ED (mg/d)**	**MMSE**	**BDI**
1	70–80	2	2	Left	550	30	5
2	70–80	4	2	Right	0	30	2
3	50–60	7	2	Right	310	30	6
4	50–60	10	2	Right	560	29	2
5	40–50	11	2	Left	1,092	29	7
6	70–80	10	2	Left	550	30	3
7	60–70	9	2	Right	1,220	28	4
8	40–50	9	2	Right	730	28	10
9	50–60	5	3	Left	580	28	17
10	70–80	6	3	Right	600	29	7
11	40–50	3	2	Left	600	30	14
12	60–70	10	2	Right	845	28	0
13	60–70	5	2	Right	500	29	3
14	20–30	10	2	Right	275	30	12
15	70–80	17	3	Left	450	28	3
16	20–30	14	1	Right	300	30	13
17	60–70	19	2	Left	240	28	1
18	60–70	4	2	Right	610	29	7
19	60–70	9	2	Left	880	24	4
20	50–60	1	1	Right	254	30	3
M ± SD	58.5 ± 14.1	12.8 ± 20.9			557 ± 297	28.9 ± 1.4	6.2 ± 4.7

*H&Y stage, Hoehn and Yahr stage; ED, equivalence dose; MMSE, Mini-Mental State Examination; BDI, Beck Depression Inventory. Units, disease duration and age in years; L-Dopa ED in mg/d. M, mean; SD, standard deviation*.

### Effects on MDS-UPDRS-III

**Figure 2:**
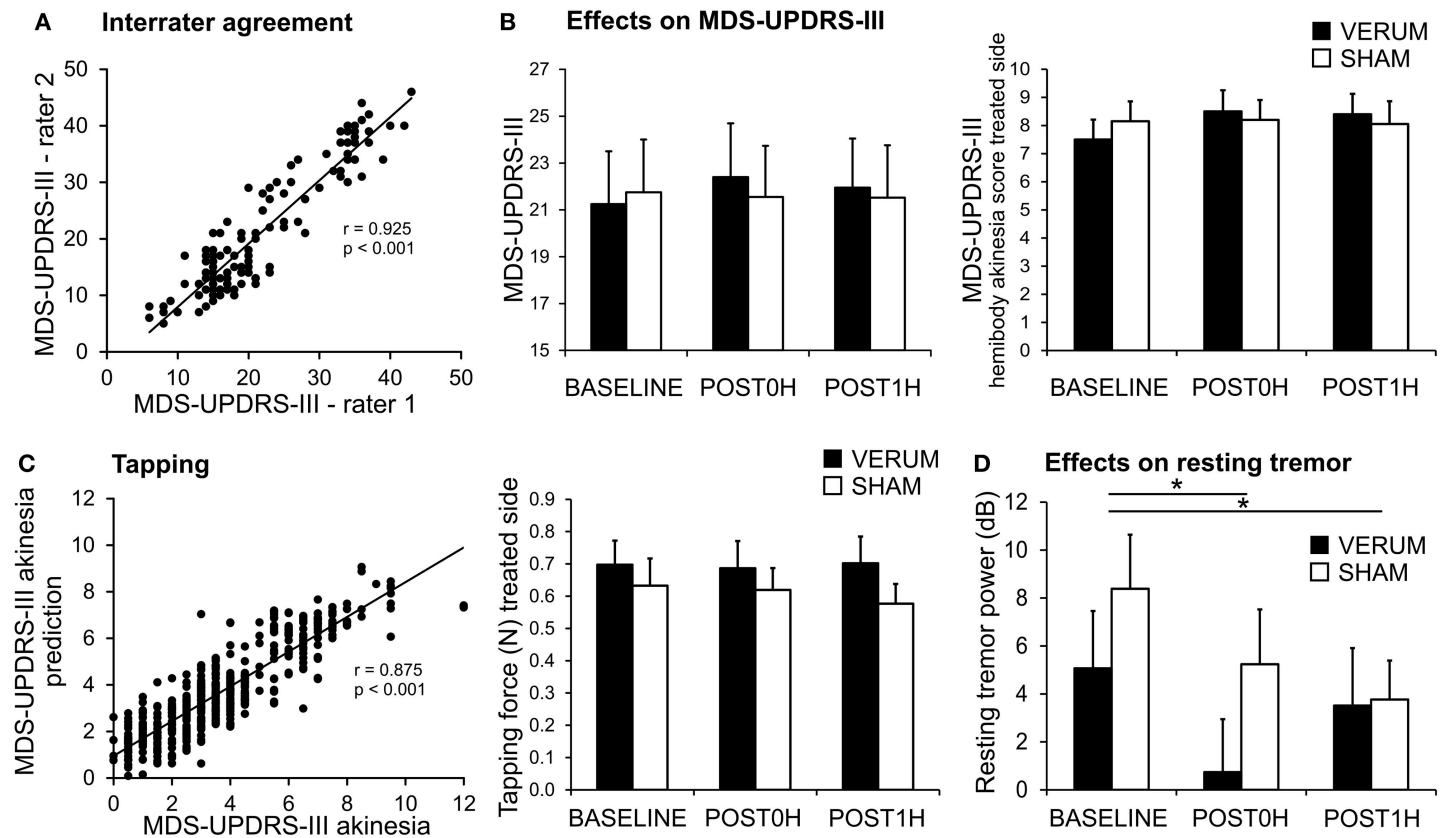
Effects of associative dual-site rTMS on PD motor symptoms. **(A)** MDS-UPDRS-III was videotaped and rated by two certified and blinded MDS-UPDRS-III raters. There was high inter-rater agreement between both raters (C.F. and T.B.W.) as demonstrated in the scatter plot. **(B)** MDS-UPDRS-III was similar for VERUM (filled bars) and SHAM (empty bars) interventions at BASELINE and did not change significantly after the stimulation (left panel). There was also no significant effect on the MDS-UPDRS-III hemibody akinesia score (sum of items 4–8) of the treated side (right panel). **(C)** MDS-UPDRS-III hand akinesia scores (sum of items 4–6) were modeled using a linear mixed model from tapping performance parameters. The model was highly predictive for the MDS-UPDRS-III hand akinesia score when employing mean tapping force, mean interval between taps, standardized tapping force and standardized interval between taps (Left). These parameters were further analyzed (see results section). Tapping force for the treated side is depicted as an example (Right). We found a significant effect for CONDITION (BASELINE, POST0H, POST1H) without an interaction with TIME. Thus, no effect of the VERUM intervention can be inferred. **(D)** Resting tremor power tended to be reduced after VERUM intervention, but it decreased significantly after both interventions (^*^p < 0.05).

Inter-rater agreement was high with respect to MDS-UPDRS-III throughout the experiments (Figure 2A, Pearson's correlation of MDS-UPDRS-III scores: *r* = 0.925 *p* < 0.001; intraclass correlation ICC(3,k) = 0.952, 95%-CI 0.931–0.967).

With respect to TMS efficacy repeated measures ANOVA revealed neither a significant main effect of CONDITION [VERUM vs. SHAM, rmANOVA, *F*_(1, 19)_ = 0.652, *p* = 0.430], nor an interaction CONDITION ^*^ TIME [*F*_(2, 38)_ = 0.872, *p* = 0.427], nor a strong numeric trend in favor of or against the VERUM intervention (Figure 2B). After normalization of POST0H and POST1H MDS-UPDRS-III scores to BASELINE we also found no significant main effect for CONDITION [VERUM vs. SHAM, rmANOVA, *F*_(1, 19)_ = 1.432, *p* = 0.246] nor a significant effect for the interaction CONDITION ^*^ TIME [*F*_(2, 38)_ = 0.071, *p* = 0.794]. POST0H and POST1H average values did not differ significantly from unity after normalization to BASELINE neither in the VERUM nor in the SHAM condition (one-sample *t*-tests, *p* ≥ 0.212, Bonferroni-corrected). Thus, VERUM stimulation had no influence on the global endpoint of the study.

Improvement in the MDS-UPDRS-III hemibody and hand akinesia scores of the treated side as well as MDS-UPDRS-III-tremor scores were assessed as lateralized endpoints. Again we found no significant effect of the intervention—hemibody akinesia score of treated side (Figure 2B): main effect of CONDITION [*F*_(1, 19)_ < 0.001, *p* = 0.999], interaction CONDITION ^*^ TIME [*F*_(2, 38)_ = 2.610, *p* = 0.087], hand akinesia score of treated side: main effect of CONDITION [*F*_(1, 19)_ = 0.092, *p* = 0.765], interaction CONDITION ^*^ TIME [*F*_(2, 38)_ = 0.267, *p* = 0.767], tremor score: main effect of CONDITION [*F*_(1, 19)_ = 3.401, *p* = 0.081], interaction CONDITION ^*^ TIME [*F*_(2, 38)_ = 1.570, *p* = 0.221].

In summary, there was no significant effect of the VERUM intervention on MDS-UPDRS-III and selected subscores.

### Effects on Tapping Performance and Spectral Power of Tremor Movements

Tapping performance was assessed as another lateralized endpoint. A mixed model analysis was used to identify tapping parameters that optimally predicted the MDS-UPDRS-III akinesia score of the corresponding arm.

The MDS-UPDRS-III hand akinesia scores were well-predicted by the model (*r*^2^ = 0.755, *r* = 0.875, *p* < 0.001; Figure 2C). Out of 8 parameters we determined (i) mean tapping force (*p* = 0.021), (ii) mean interval between taps (*p* = 0.034), (iii) standardized tapping force (*p* = 0.007), and (iv) standardized tapping interval (*p* < 0.001) as significant for MDS-UPDRS-III prediction. The effect of ADS-rTMS intervention on this set of informative parameters was then evaluated, for the treated hand only, using repeated measures ANOVA. For the mean tapping force, there was a trend for CONDITION [*F*_(1, 18)_ = 4.409, *p* = 0.050], but no interaction CONDITION ^*^ TIME [*F*_(2, 36)_ = 0.536, *p* = 0.590; Figure 2C]. The effect of CONDITION was driven by a slightly higher tapping force throughout the day of the VERUM intervention. For the remaining parameters, we found neither a significant main effect of CONDITION [*F*_(1, 18)_ ≤ 2.182, *p* ≥ 0.157] nor an interaction CONDITION ^*^ TIME [*F*_(2, 36)_ ≤ 0.817, *p* ≥ 0.450].

Resting and postural tremor power were also evaluated as lateralized endpoints (Figure 2D). For resting tremor there was a significant main effect for spectral power of the treated hand for CONDITION [*F*_(1, 18)_ = 7.541, *p* = 0.013] and TIME [*F*_(2, 36)_ = 6.111, *p* = 0.005], but no significant CONDITION ^*^ TIME interaction [*F*_(2, 36)_ = 1.686, *p* = 0.200]. The effect for CONDITION was driven by a lower spectral power after VERUM intervention (*p* = 0.031, uncorrected, Figure 2D), while the effect of TIME was driven by a lower spectral power at POST0H and POST1H (*p* ≤ 0.045, Bonferroni-corrected). For postural tremor we found no main effect of CONDITION [*F*_(1, 18)_ = 1.321, *p* = 0.265] nor for the interaction CONDITION ^*^ TIME [*F*_(2, 36)_ = 3.070, *p* = 0.059], but again a significant main effect of TIME [*F*_(2, 36)_ = 10.305, *p* < 0.001]. *Post-hoc t*-tests revealed that the main effect of time was driven by a significant decline in spectral tremor power following the intervention at POST0H and POST1H for both conditions (*p* ≤ 0.024, Bonferroni-corrected). As there was no significant interaction CONDITION ^*^ TIME we interpret the decrease in tremor power following both interventions as an unspecific effect (e.g., anxiety before the intervention).

In summary, there were neither meaningful beneficial nor detrimental effects of the intervention on either tapping performance or tremor.

We conducted additional experiments involving stimulation of M1 and either SMA or M1+50 as a premotor site as detailed in the Supplementary Material. These interventions did not yield any beneficial effect either (for details, see Supplementary Material).

## Discussion

We designed a TMS intervention aiming to ameliorate Parkinsonian motor symptoms by employing principles of associative stimulation. The protocol was well-tolerated. None of the tested variants of this stimulation protocol had any significant impact on motor parameters. Our experimental strategy was based on a variety of assumptions. Below, we examine possible violations of these assumptions and additional reasons explaining why results were negative, and outline consequences for future attempts of non-invasive treatment protocols.

The anatomical basis for a short latency effect of motor cortical stimulation on STN neurons is the presence of a hyperdirect tract connecting cortex and STN monosynaptically. This tract has been shown to exist in animal studies (18) and there is increasing evidence of a hyperdirect tract in humans (36–38). A small number of studies showed that TMS directed to motor cortical areas induces STN activity (22, 23). The ability to activate this tract may, on the other hand, be compromised in PD patients as there is evidence for some degree of degeneration in the tract (39).

Little is known how cortico-basal ganglia projections may be specifically activated by TMS and how they would influence individual STN neurons. Fibers originating from SMA or PMd (18, 40, 41) may predominantly terminate in non-motor subregions within STN instead of motor regions. TMS pulses were intended to induce co-activation in a group of STN neurons. Although stimulation intensities near the motor threshold have been shown to induce volleys in descending fibers (35), stimulation intensities may have been too low to modulate the activity of a sufficiently large number of neurons, or to generate action potentials in cortico-fugal projection neurons targeting the STN in particular. Previous studies also successfully employed subthreshold TMS in PD patients (17, 42) and variably achieved beneficial effects in single sessions (43, 44) or only after multiple days of treatment (45, 46). Therefore, effects may be present after a first session but may also become apparent only after repeated applications. Hence we cannot exclude the possibility that ADS-rTMS might have been effective if higher stimulation intensities or multiple sessions had been used.

The interstimulus interval (ISI) of 25 ms used in our TMS protocol was based on the theoretical assumption that pathogenic oscillations are present at about 20 Hz. However, the relevant beta oscillations may peak at any frequency between 15 and 30 Hz (33, 47) or exhibit even two peaks at distinct frequencies (48). Therefore, an ISI of 25 ms may have been less effective to desynchronize STN neurons. Because we had no means of assessing individual beta oscillations in STN, it was not possible to individually adjust the ISI for optimal effects. Furthermore, studies using PAS found that synaptic plasticity may be deficient in the absence of dopaminergic medication in the motor cortex of patients with PD (49, 50). This has been recently shown to correlate with motor performance and be in part reversible by dopamine replacement (51), suggesting not only a pathophysiological link between plasticity and dopamine availability, but also between motor cortical plasticity and akinesia in PD. The human STN receives dopaminergic projections from midbrain dopamine neurons (52). Studies in rat striatal slices have shown dopamine to be an essential component of activity-dependent synaptic plasticity at the input to the basal ganglia (53). Therefore, overnight withdrawal of dopaminergic medication in the present study may have compromised the ability of neuronal synapses in the STN to undergo long-term depression. On the other hand, Shirota et al. (46), Strafella et al. (54) and Strafella et al. (55) have shown that TMS delivered to a single cortical site, if anything, may facilitate striatal dopamine release. Therefore, ADS-rTMS is unlikely to have augmented the dopamine deficiency induced by overnight withdrawal of dopaminergic medication.

Whether the intended cortical targets have been activated remains another possible area of uncertainty. PMd or SMA stimulation effects cannot be verified physiologically as easily as M1 effects by assessing MEPs. Additionally, physiological localization of PMd is not trivial as evidenced by the considerable heterogeneity with respect to PMd stimulation sites used in previous studies. In the present study we employed TMS mapping which yielded a possible PMd site 32 mm anterior to M1 (M1+32). This site is near a PMd site at 25 mm anterior of M1 used previously (56–59). More precisely, M1+32 was based on the absence of significant known effects tied to M1 conditioning and on suggestions of a physiological effect of conditioning stimulation on M1 excitability whose timing (at 23 ms) would be consistent with latencies of effects on M1 excitability observed in STN-DBS (60) suggesting subcortical processing. Civardi et al. (61) also described conditioning effects at M1+50 mm which we tested in an additional experiment. In line with reports of another group (62) we could not replicate the described physiological effects, neither did we find any clinical effect on PD symptoms at this stimulation site. SMA stimulation proved difficult due to its deep location in the interhemispheric fissure as we found that even maximal stimulation intensities were insufficient to reliably activate the leg-associated motor area in 2 participants.

Despite the fact that the present study failed to reach a clinical improvement, we believe that it may stimulate future attempts at non-invasive treatment of PD by targeting pathogenic oscillations at subcortical targets. Apart from the limitations discussed above, our study has certain strengths that may inform the design of future intervention trials: The assessment of PD symptoms was based on randomized videography of the MDS-UPDRS-III and on objective parameters. This ensured that researcher bias was minimized. Furthermore, a novel coil design enabled us to stimulate two cortical areas located very close to each other.

## Conclusions

In summary, associative dual-site rTMS did not generate a clinically meaningful beneficial effect on Parkinsonian motor symptoms. The present findings leave us with a very large number of TMS parameters and other parameters to be optimized. Although future information may help to constrain this vast space, a more promising strategy may consist in estimating parameters individually with optimized parameter estimation paradigms (e.g., Bayesian optimization) and on brain-state markers of PD pathology as potentially accessible from EEG.

## Ethics Statement

All subjects gave written informed consent in accordance with the Declaration of Helsinki. The protocol was approved by the Ethics Committee of the University of Leipzig, file-no.: 351-13-26082013.

## Author Contributions

CF: conception, fund raising, data acquisition, data analysis, writing of the manuscript. CD: data acquisition, data analysis, writing of the manuscript. TW: data acquisition, data analysis, writing of the manuscript. JH-H: patient recruitment, data acquisition. J-JR: conception, data acquisition. DW: conception, data acquisition. JC: conception, fund raising, data analysis, writing of the manuscript. All authors approved the final version of the manuscript.

### Conflict of Interest Statement

The authors declare that the research was conducted in the absence of any commercial or financial relationships that could be construed as a potential conflict of interest.
