# Supplementary material for: Dual-Site Transcranial Magnetic Stimulation for the Treatment of Parkinson's Disease
Source: Front Neurol. 2019 Mar 7;10:174. doi: 10.3389/fneur.2019.00174 (PMC6417396; doi:10.3389/fneur.2019.00174)
Supplement: Supplementary file 1 [file Data_Sheet_1.docx]

**Dual-site Transcranial Magnetic Stimulation for the Treatment of Parkinson’s Disease – Supplementary material**

**Tapping and tremor data preprocessing**

Tapping data was sampled at 1000 Hz using a CED Micro1401 MK2 (Cambridge electronic design Ltd., Milton, UK). Signals were detrended using a 0.5 Hz 4^nd^ order Butterworth high-pass filter, then filtered with a 40 Hz 4^nd^ order Butterworth low-pass filter. Afterwards they were locally differentiated and rectified and peak detection was used to determine the location of positive peaks corresponding to performed taps. The amplitude of each tap was then determined from the non-differentiated and non-rectified signal and scaled to Newton (N). For this we previously recorded calibration curves for the force transducer using a commercial standard weight of 1 kg. The following characteristics of the finger tapping performance were determined for each tapping experiment 1) mean force of taps, 2) standard deviation of tapping force, 3) standardized force of taps (mean divided by standard deviation of tapping force), 4) a decrement metric represented by the slope of tap force over time, 5) mean interval between taps, 6) standard deviation of intervals, 7) standardized interval between taps (mean interval divided by standard deviation of intervals) and 8) a decrement metric represented by the slope of intervals over time. The standardized parameters (no. 3 and 7) are markers for the uniformity of the respective parameter.

Tremor data was recorded at 500 Hz, detrended using a 0.5 Hz 4^nd^ order Butterworth high-pass filter, then filtered with a 40 Hz 4^nd^ order Butterworth low-pass filter. Then, data was cut to contain 20 seconds of either resting or holding data and the latest parts of the rest phase were used where no movement artefacts (from raising the hands to hold position) were discernible. We then applied Welch’s method for frequency-power-analysis (Matlab function *pwelch*) and extracted the peak tremor frequency.

**PMd localizer experiment**

**Methods**

The dorsal premotor cortex (PMd) was chosen as cortical target outside of M1 because of its direct projections to the STN (1) and because of its location close to the skull surface allowing for easy accessibility. Published coordinates for PMd stimulation vary widely between 8 and 50 mm anterior to M1 (mostly 25 mm, see (2–7). In addition, the novel geometry of D-shaped coils necessitated establishing the optimal placement of the premotor coil empirically due to missing experience. To account for these facts, we conducted a TMS experiments to locate a dorsal premotor stimulation site: 1) We reasoned that the transition between M1 and PMd should be at, or close to, the nearest coil position anterior to M1 from which no effects on intrinsic M1 circuits (as evidenced by modulation of MEP amplitude and short interval cortical inhibition - SICI) could be detected. 2) We aimed to investigate whether PMd stimulation had effects on M1 excitability at latencies sufficiently long to indicate basal ganglia processing. These localizer experiments were conducted in healthy subjects. Exclusion criteria were: participation in other studies and known contraindications to TMS (8), especially epilepsy, medication with antidepressants, neuroleptics, benzodiazepines or antibiotics and presence of implanted devices or metal near the head.

Probing M1-to-PMd transition: Nineteen healthy subjects (age 25.9 ± 4.4 years; 12 male, 7 female) participated in the experiment. The location of M1 was defined physiologically. The hot spot for stimulation of the abductor pollicis brevis muscle (APB) was identified employing low frequency (< 0.2 Hz) stimulation at multiple sites supposedly overlying M1 while recording MEPs using surface EMG from the APB. We used one D-shaped coil for stimulation of M1 (test coil) which was oriented as to induce a current flow in a sagittal direction within the brain. After finding a suitable hot spot, the resting motor threshold of the APB (APB-RMT) was determined using threshold hunting (9). Thereafter, a second D-shaped coil (“conditioning coil”) was positioned “head to head” to the test coil with the current direction inverted (see also Fig. 1B). The conditioning coil was moved in a sagittal direction to also identify the APB-hotspot. This spot was labelled “M1+0”, as it corresponded to a location 0 mm anterior (and therefore being equal) to M1. For this coil APB-RMT was determined separately. M1+x stimulation points are defined as lying x mm anterior to M1 in a sagittal direction. We tested conditioning with ISIs of 2, 4, 6 and 8 ms over locations M1+0, M1+10, M1+20, M1+28, M1+36, M1+44 and M1+52. At each stimulation site 10 test pulses were applied for every ISI in a randomized order; the order of stimulation sites was chosen randomly. The conditioning pulse was applied through the conditioning coil using a stimulation strength of 80% APB-RMT (optimal for induction of SICI, (10), the test stimulus over the test coil with 120% APB-RMT. Additionally, we applied 10 single pulse stimuli with 120% over each M1+x location (with the conditioning coil) as well as over the M1 location (with the test coil). Apart from analyzing modulatory effects of conditioning TMS at each M1+x side we also used an interpolation based on piecewise cubic Hermite interpolating polynomials (implemented in Matlab function *pchip*) to better visualize M1 to PMd transition (Supplem. Fig. 1A).

Longer-latency interactions (14-38 ms) consistent with PMd stimulation: Following experiment 1 we tested longer latency ISIs at 14, 17, 20, 23, 26, 29, 32, 35 and 38 ms at the site where conditioning TMS effects on M1 were no longer detectable, which presumably overlay PMd. Nineteen healthy subjects (age 25.4 ± 4.3 years; 9 male, 10 female) participated in this experiment. Stimulation intensity was 95% or 120% APB-RMT for the conditioning and 120% for the test stimulus. 95% APB-RMT was chosen to resemble the stimulation intensity used later during the treatment experiment, 120% APB-RMT was used to maximize the chance of finding a conditioning effect dependent on suprathreshold stimulation.

The rationale for conducting this experiment is that a premotor stimulus may project to the STN via the hyperdirect tract where it may influence basal ganglia processing which in result should have an impact on M1 excitablity. The latter has been shown in a previous study (11) where MEP facilitation stimulating M1 followed a single pulse of current delivered through electrodes placed in the STN at latencies of 21 – 24 ms. This latency is considerably longer than what would be expected for direct antidromic activation of the hyperdirect tract and therefore indicates basal ganglia processing. We rationed that if a similar activation could be evoked by activity induced in STN by cortical stimulation via fast-conducting hyperdirect cortico-subthalamic projections, then a conditioning effect following basal ganglia processing would become visible at ISIs around 23 - 26 ms implying a latency of about 2-3 ms resulting from activation of the hyperdirect tract to the STN (12).

In both experiments MEPs were amplified with a D360 amplifier (Digitimer Ltd, Welwyn Garden City, UK) and at 2000 Hz using a CED Micro1401 MK2 (Cambridge electronic design Ltd., Milton, UK). Data was band-pass filtered with a 4^th^ order Butterworth filter (0.1 – 500 Hz) and cut to encompass a time window of 300 ms starting from the test pulse. The peak-to-peak MEP amplitude was calculated in each trial. MEP peak-to-peak amplitudes below 50 µV were set to 0 µV. For each subject MEP amplitudes for any of the given condition (that is ISI and stimulation site) were averaged and conditioning effects were calculated by dividing mean MEPs generated in conditioning trials by mean MEPs generated in M1-only single pulse trials. Statistical significance was estimated based on comparison of the normalized values against unity (one-sample t-tests) for tested ISIs and stimulation sites.

**Results**

In the first part of the PMd localizer experiment we tested short-latency interactions using ISIs of 2, 4, 6 and 8 ms with conditioning pulses applied to 7 positions above or anterior to M1 in 19 healthy subjects (age 25.9 ± 4.4 years, 12 male, 7 female). Amplitudes of single pulse-evoked MEPs were not different when stimulating above M1 with suprathreshold pulses using either the coil used for conditioning (at M1+0, identical to M1 position) or the test coil (at M1, p = 0.201). We found a strong inhibitory (SICI) effect for ISIs of 2 – 4 ms, exclusively for conditioning at M1+0, M1+10, M1+20 and M1+28 (one-sample t-test vs. 1, p < 0.029, Bonferroni-corrected, Supplem. Fig. 1A). Between M1+28 and M1+36 single pulse MEP amplitude decreased progressively with more anterior stimulation positions (Supplem. Fig. 1B). Interpolation suggested absence of detectable intrinsic SICI-like M1 effects at conditioning coil positions anterior to M1+28. Based on this finding and previously reported PMd stimulation sites (5–7, 13) we used a stimulation site at 32 mm anterior of M1 (M1+32) as premotor area for subsequent experiments. In the second part of the localizer experiment we investigated modulatory effects of M1+32 conditioning on MEPs evoked at M1, using ISIs of 14 to 38 ms in steps of 3 ms and conditioning at 95% and 120% APB-RMT. Nineteen healthy subjects (age 25.4 ± 4.3 years, 9 male, 10 female) participated in this experiment. Application of test stimuli over M1 revealed robust facilitation for conditioning over M1 with 95% APB-RMT at ISIs of 17 and 20 ms (paired t-test against unity, p < 0.046, Bonferroni-corrected; for ISI of 14 – 26 and 32 ms p < 0.044 uncorrected, Supplem. Fig. 1C, left panel), most likely corresponding to intracortical facilitation (ICF) described previously for these ISIs (10, 14). By contrast, we found no significant effect of conditioning at M1+32 at the probed latencies, neither for conditioning with 95% nor with 120% APB-RMT applying Bonferroni-correction. For conditioning with 95% APB-RMT we found a trend for ISIs of 14 and 23 ms (paired t-test, p < 0.034 uncorrected, Supplem. Fig. 1C, left panel). These missing MEP conditioning effects at M1+32, together with the negligible amplitude of single pulse stimulation at 120% and 95% APB-RMT (Supplem. Fig. 1C, right panel), suggested the absence of detectable intrinsic M1 stimulation effects and, therefore, physiologically selective premotor stimulation. However, at ISIs of 23-26 ms no modulatory effects indicative of basal ganglia processing of TMS pulses could be detected, neither for M1 nor for M1+32 conditioning.

**Additional experiments at different cortical locations**

**Methods**

In addition to the main experiment involving M1+32 stimulation we conducted two experiments with a more limited number of PD patients involving other stimulation sites anterior to the primary motor cortex as one component of the dual-site paradigm: (i) Civardi and colleagues described a premotor-motor interaction for a PMd site at 50 mm anterior of M1 (2). We tested the clinical effect of stimulation at this point (M1+50). (ii) The location of the supplementary motor area (SMA) was derived from a previous study (15, 16) where its stimulation was found to be effective for treatment of PD symptoms. To target the SMA, we first determined the location of the leg area in M1 recording MEPs from the contralateral tibialis anterior muscle (TA). At the M1-hotspot for TA muscle activation, we determined the RMT using threshold hunting. Then the TMS coil was moved in an anterior direction while stimulating at 120% of the TA-RMT until we did not record a MEP from TA anymore (17, 18). VERUM stimulation over SMA was applied at 95% TA-RMT and SHAM stimulation at 20% TA-RMT. We recorded and analyzed MDS-UDPRS-III for both additional experiments as described above for dual-site stimulation at M1 and M1+32.

**Results**

We conducted additional experiments using dual-site associative stimulation over M1+50/M1 and SMA/M1 with a smaller number of PD patients.

We included 9 patients (age 61.1 ± 9.6 years, 6 male, 3 female) in an experiment involving M1+50/M1 stimulation. We found no significant main effect of CONDITION (VERUM vs. SHAM, rmANOVA, F(1,8) = 3.360, p = 0.104) nor an interaction CONDITION * TIME (F(2,16) = 0.027, p = 0.973) for MDS-UPDRS-III sum score. Furthermore, we found neither a significant main effect nor an interaction for the MDS-UPDRS-III akinesia score of the treated hand (main effect of CONDITION, VERUM vs. SHAM, rmANOVA, F(1,8) = 3.306, p = 0.107; interaction CONDITION * TIME, F(2,16) = 1.304, p = 0.299).

For SMA/M1 we included 11 patients. For 2 patients we were not able to find a hot spot for the anterior tibialis muscle using the novel coils. Therefore, 9 patients (age 58.8 ± 10.1 years, 5 male, 3 female) eventually were able to receive the ADS-rTMS intervention. We found no significant main effect of CONDITION (VERUM vs. SHAM, rmANOVA, F(1,6) = 2.050, p = 0.202) nor an interaction CONDITION * TIME (F(2,12) = 1.201, p = 0.335) for MDS-UPDRS-III sum score. Similarly, there was no significant main effect nor an interaction for the MDS-UPDRS-III akinesia score of the treated hand (main effect of CONDITION, VERUM vs. SHAM, rmANOVA, F(1,6) = 3.857, p = 0.097; interaction CONDITION * TIME, F(2,14) = 0.137, p = 0.874).

**Figure Legends**

**Suppl. Figure 1:** *Premotor site localization.* A) Effects of short latency interaction effects of subthreshold conditioning at multiple sites anterior of M1 and for multiple interstimulus intervals (ISI) were investigated and results are depicted as graphs. A short interval cortical inhibition (SICI) was seen at M1 up to 28 mm anterior of M1. We found no other significant conditioning effect at short latencies. A piecewise cubic Hermite interpolating polynomial was used to interpolate between sites and ISIs not measured to better assess M1 to premotor transition. The results of this interpolation is depicted in the center of the panel with markings for where the measured graphs are to be associated to, where SICI effects are significant at different thresholds (yellow and blue lines for p < 0.01 and p < 0.001 thresholds) and where from this premotor to motor transition can be derived (blueish area). The interpolation is also depicted in each sub panel as a dotted line. B) Relative MEP amplitude for suprathreshold TMS at multiple sites anterior of M1. MEP amplitude vanishes anterior of the 28 mm point. C) Long latency effects were investigated for conditioning at M1 as well as a premotor site which we defined from loss-of-significance at panels A and B to be at 32 mm (M1+32). We found intracortical facilitation (ICF) for conditioning at M1 using near-threshold stimulation but we found no effect for neither near- nor suprathreshold conditioning at M1+32. D) Suprathreshold single pulses yielded large MEPs only at M1 while near-threshold stimulation and M1+32 suprathreshold stimulation resulted in similar missing or only very low MEPs. No MEPs were recorded using near-threshold TMS of M1+32 (* p < 0.05, ** p < 0.01, *** p < 0.001).

REFERENCES

1. Nambu A, Tokuno H, Takada M. Functional significance of the cortico-subthalamo-pallidal 'hyperdirect' pathway. *Neurosci Res* (2002) **43**(2):111–7.

2. Civardi C, Cantello R, Asselman P, Rothwell JC. Transcranial magnetic stimulation can be used to test connections to primary motor areas from frontal and medial cortex in humans. *Neuroimage* (2001) **14**(6):1444–53. doi:10.1006/nimg.2001.0918

3. Picard N, Strick PL. Imaging the premotor areas. *Curr Opin Neurobiol* (2001) **11**(6):663–72.

4. Bäumer T, Schippling S, Kroeger J, Zittel S, Koch G, Thomalla G, et al. Inhibitory and facilitatory connectivity from ventral premotor to primary motor cortex in healthy humans at rest--a bifocal TMS study. *Clin Neurophysiol* (2009) **120**(9):1724–31. doi:10.1016/j.clinph.2009.07.035

5. Groppa S, Schlaak BH, Münchau A, Werner-Petroll N, Dünnweber J, Bäumer T, et al. The human dorsal premotor cortex facilitates the excitability of ipsilateral primary motor cortex via a short latency cortico-cortical route. *Hum Brain Mapp* (2012) **33**(2):419–30. doi:10.1002/hbm.21221

6. Ni Z, Isayama R, Castillo G, Gunraj C, Saha U, Chen R. Reduced dorsal premotor cortex and primary motor cortex connectivity in older adults. *Neurobiol Aging* (2015) **36**(1):301–3. doi:10.1016/j.neurobiolaging.2014.08.017

7. Fiori F, Chiappini E, Candidi M, Romei V, Borgomaneri S, Avenanti A. Long-latency interhemispheric interactions between motor-related areas and the primary motor cortex: a dual site TMS study. *Sci Rep* (2017) **7**(1):14936. doi:10.1038/s41598-017-13708-2

8. Rossi S, Hallett M, Rossini PM, Pascual-Leone A. Safety, ethical considerations, and application guidelines for the use of transcranial magnetic stimulation in clinical practice and research. *Clinical Neurophysiology* (2009) **120**(12):2008–39. doi:10.1016/j.clinph.2009.08.016

9. Silbert BI, Patterson HI, Pevcic DD, Windnagel KA, Thickbroom GW. A comparison of relative-frequency and threshold-hunting methods to determine stimulus intensity in transcranial magnetic stimulation. *Clin Neurophysiol* (2013) **124**(4):708–12. doi:10.1016/j.clinph.2012.09.018

10. Kujirai T, Caramia MD, Rothwell JC, Day BL, Thompson PD, Ferbert A, et al. Corticocortical inhibition in human motor cortex. *J Physiol (Lond )* (1993) **471**:501–19.

11. Kuriakose R, Saha U, Castillo G, Udupa K, Ni Z, Gunraj C, et al. The nature and time course of cortical activation following subthalamic stimulation in Parkinson's disease. *Cereb Cortex* (2010) **20**(8):1926–36. doi:10.1093/cercor/bhp269

12. Miocinovic S, Hemptinne C de, Chen W, Isbaine F, Willie JT, Ostrem JL, et al. Cortical Potentials Evoked by Subthalamic Stimulation Demonstrate a Short Latency Hyperdirect Pathway in Humans. *J Neurosci* (2018) **38**(43):9129–41. doi:10.1523/JNEUROSCI.1327-18.2018

13. Ni Z, Gunraj C, Nelson AJ, Yeh I-J, Castillo G, Hoque T, et al. Two phases of interhemispheric inhibition between motor related cortical areas and the primary motor cortex in human. *Cereb Cortex* (2009) **19**(7):1654–65. doi:10.1093/cercor/bhn201

14. Vucic S, Howells J, Trevillion L, Kiernan MC. Assessment of cortical excitability using threshold tracking techniques. *Muscle Nerve* (2006) **33**(4):477–86. doi:10.1002/mus.20481

15. Hamada M, Ugawa Y, Tsuji S. High-frequency rTMS over the supplementary motor area for treatment of Parkinson's disease. *Mov Disord* (2008) **23**(11):1524–31. doi:10.1002/mds.22168

16. Shirota Y, Ohtsu H, Hamada M, Enomoto H, Ugawa Y. Supplementary motor area stimulation for Parkinson disease: a randomized controlled study. *Neurology* (2013) **80**(15):1400–5. doi:10.1212/WNL.0b013e31828c2f66

17. Gregori B, Currà A, Dinapoli L, Bologna M, Accornero N, Berardelli A. The timing and intensity of transcranial magnetic stimulation, and the scalp site stimulated, as variables influencing motor sequence performance in healthy subjects. *Exp Brain Res* (2005) **166**(1):43–55. doi:10.1007/s00221-005-2337-3

18. Doyle Gaynor L, Kühn AA, Dileone M, Litvak V, Eusebio A, Pogosyan A, et al. Suppression of beta oscillations in the subthalamic nucleus following cortical stimulation in humans. *Eur J Neurosci* (2008) **28**(8):1686–95. doi:10.1111/j.1460-9568.2008.06363.x
