# Supplementary figures and images for: Dual-Site Transcranial Magnetic Stimulation for the Treatment of Parkinson's Disease
Source: Front Neurol. 2019 Mar 7;10:174. doi: 10.3389/fneur.2019.00174 (PMC6417396; doi:10.3389/fneur.2019.00174)

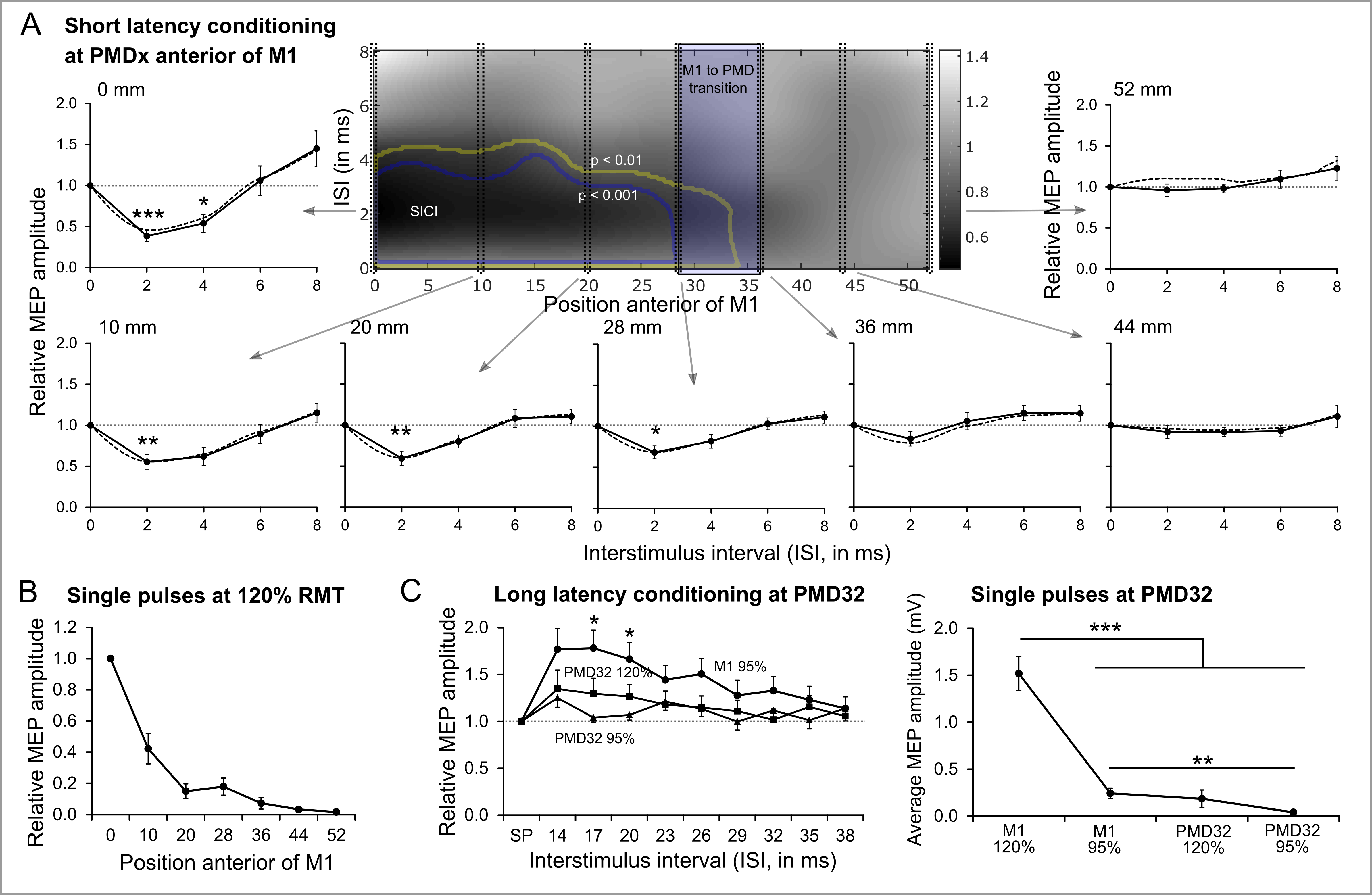

Supplement: Supplementary file 2 [file Image_1.TIF]
